# Active school transport in an urban environment:prevalence and perceived barriers

**DOI:** 10.1186/s12889-023-15464-7

**Published:** 2023-03-23

**Authors:** Isabel Wex, Mandy Geserick, Tim Leibert, Ulrike Igel, Carolin Sobek, Christof Meigen, Wieland Kiess, Mandy Vogel

**Affiliations:** 1 LIFE Child, LIFE Leipzig Research Center for Civilization Diseases, Leipzig University, Leipzig, Germany; 2 Department of Women and Child Health, Hospital for Children and Adolescents and Center for Paediatric Research (CPL), Leipzig University, Leipzig, Germany; 3 Leibniz Institute for Regional Geography, Leipzig, Germany; 4 Department of Social Work, University of Applied Science, 99085 Erfurt, Germany

**Keywords:** Active school transport, Perceived barriers, Physical activity

## Abstract

**Background:**

Active school transport (AST) can increase children’s and adolescents’ physical activity. The proportion of children and adolescents who engage in AST has declined internationally in recent decades. This study examines the prevalence, correlates, and perceived barriers to AST in the city of Leipzig, Germany.

**Methods:**

The study sample includes 1070 participants, 364 children and 706 adolescents, aged between 6 and 18 years, as well as their parents. The parents as well as adolescents age 10 and above completed questionnaires concerning sociodemographic variables, means of transport/AST and perceived barriers to AST. The distance between home and school was calculated as the network distance from the home to school address using the Dijkstra algorithm. Based on these data, logistic models were fitted in a two-step variable selection process, using AST as the dependent variable.

**Results:**

Approximately half of the children (59%) and adolescents (51%) engaged in AST. The prevalence of AST exhibited a negative correlation with age (Odds Ratio (OR) = 0.94, 95% confidence interval (CI) = 0.9–0.99, *p* = 0.015) and did not significantly differ by gender (children: OR_girls_ = 1.5, CI = 0.95–2.25, *p* = 0.075, adolescents: OR_girls_ = 1.01, CI = 0.75–1.37, *p* = 0.924). A high socioeconomic status was positively correlated to AST on the morning trip (OR = 1.7, CI 1.3–2.21, *p* < 0.01) but negatively on the afternoon trip (OR = 0.7, CI = 0.53–0.9, *p* < 0.01) in the summer. Common barriers for children (from their parents’ perspective) and for adolescents (from their own and their parents’ perspective) were *distance* and a *heavy load to carry*. The parents of adolescents did not perceive any other specific barriers as a serious impediment. Further significant barriers perceived by the younger children’s parents were adults giving a lift on the way to other errands, no other children to walk or cycle with, and too much traffic. Too much traffic was also a significant barrier for adolescents, as were taking too much time and bad weather conditions.

**Conclusions:**

Future interventions promoting AST in an urban environment should be guided by the identified perceived barriers.

**Trial registration:**

LIFE Child has been retrospectively registered with ClinicalTrials.gov (NCT02550236).

**Supplementary Information:**

The online version contains supplementary material available at 10.1186/s12889-023-15464-7.

## Introduction

Physical activity is considered a key factor in the prevention of several chronic conditions [1]. However, most German children and adolescents do not meet the recommended daily 60 min of moderate-to-vigorous physical activity [2]. One opportunity to increase children’s and adolescents’ physical activity is commuting to and from school actively. Active school transport (AST) is beneficial in several ways: It is associated with better cardiovascular fitness [3], significantly higher levels of psychosocial wellbeing [4, 5], and fewer depressive symptoms [6]. On the other hand, areas around schools can be dangerous places for pedestrians and cyclists because so many parents drive their children to school, causing heavy traffic and congestion [7]. By driving their children to school by car each day, parents not only deny possible health benefits to their children on an individual level, but also contribute to environmental damage affecting the whole population. As transport accounts for around one fifth of German CO_2_ emissions, AST can counteract climate change, which is also a threat to children’s and adolescents’ health and wellbeing [8, 9]. Despite all these facts, the proportion of pupils who engage in active school transport has been declining internationally in recent decades [10–15]. In Germany, there was a decline from 84.4% in 2003 to 78.3% in 2017 [14].

An emerging body of literature discusses factors influencing AST on an individual, cultural, social, physical, and political level. Social-ecological models highlight the role of the environment in behavioral choices [16]. The environment can be assessed via objective measures, e.g., using geographic information systems, but also via measures of subject perception. There is evidence that the associations between behavior and subjective perceptions are stronger than those with objectively measurable environment parameters [17]. Therefore, subjectively perceived barriers to AST are of particular interest. In AST research, perceived barriers are defined as a person’s perceived level of challenges stemming from intrapersonal, interpersonal, environmental, and policy obstacles [18]. A summary of studies comparing barriers to active school transport perceived by children or adolescents and their parents is given in Additional Table 1. Several reviews have found that the prevalence of AST and its influencing factors vary between geographic contexts [19, 20]. However, there are also common factors across settings. For example, most studies confirm that the distance between home and school plays a decisive role [10, 21, 22]. The majority of the literature on AST originates in English-speaking countries. For Germany, there is a research gap regarding children’s and adolescents’ mobility [23]. To our knowledge, no research has previously been conducted on children’s or adolescents’ perceptions of barriers to AST in Germany.

**Table 1:** List of questions regarding the means of transport (in the adolescents’ self-report questionnaire)

1 We would like to know how you get to school and back home from school on most days! Please describe your path between the door of your home and school in summer and in winter Please give the most frequent variant, which can also consist of multiple means of transport (e.g. partly walking and partly taking a bus)!	
My most frequent way to school in summer consists of…	a walk
	a ride by bike, scooter or longboard
	a journey by car, moped or motor scooter
	a journey by bus, train or tramway
**2 Subsequently, the time spent in each transportation mode was asked for**	

Therefore, our study aimed to examine the prevalence, correlates, and perceived barriers to AST in Leipzig, a city in Germany. We analyzed which environmental characteristics and which conditions are perceived as barriers by parents of primary and secondary school pupils and adolescents themselves (age 10 and above). In addition, we examined the potential effect modifiers of age, gender, socioeconomic status (SES), and distance.

## Methods

### Study design

The present study is part of the LIFE Child study conducted at the Research Center for Civilization Diseases in Leipzig (Germany). LIFE Child is a longitudinal cohort study investigating the development of children and adolescents from pregnancy until early adulthood with a particular interest in the origins of non-communicable diseases. The annual visits comprise a comprehensive physical examination, collection of bio-samples, and several questionnaires regarding sociodemographic, psychological, and lifestyle characteristics [24, 25]. The questionnaires are completed in the study clinic using electronic case report forms.

The following analysis is based on cross-sectional data collected between September 2018 and February 2020. In cases of multiple visits per child, the most recent visit was chosen.

LIFE Child is conducted in accordance with the Helsinki Declaration and was approved by the Ethics Committee of the Medical Faculty of the Leipzig University (Reg. No. 264–10-19,042,010). All parents gave written informed consent for them and their children to be included in the study. An additional written informed consent was obtained from adolescents age 12 and above.

### Study population

The initial sample consisted of 1456 children and adolescents. 386 participants were excluded because of missing information on their means of transport (*n* = 385) or implausible values (*n* = 1). The final sample included 1070 children (*n* = 364) and adolescents (*n* = 706) aged between 6.12 and 17.97 years (mean: 11.64, standard deviation (Standart Deviation (SD): 3.09). 10 years is the age at which most children change from primary to secondary school in Germany [26]. It was therefore defined as the cut-off between children (≤ 10) and adolescents (> 10 years).

Fifty-three percent of the participants were boys. Most participants had a mid-level socioeconomic status (SES) (58%); low SES (2%) was under- and high SES overrepresented (34%). Information on SES was missing in 6% of cases. A more detailed description of participants’ socioeconomic status is provided in Additional Table 2.

**Table 2:** Questions regarding perceived barriers with answer options (multiple choice)

It is difficult for me / for my child to walk or to bike to school independently (without the company of an adult) because
Physical environment
• It is too far
• It takes too much time
• The route is too hilly/exhausting
• There are no sidewalks or bike paths
• The route is too monotonous/boring
• The route is badly illuminated
Safety
• The route is too dangerous
• It is too dangerous because of crime (strangers, gangs, drugs)
• There is too much traffic on the route
Social
• There are no other children walking or going by bike
• I am / my child is bullied, annoyed or harassed
Individual/Family preferences
• I do not have confidence in myself/ My child does not have the confidence to go
independently
• I have too heavy a load to carry./My child has too heavy a load to carry
• An adult gives me a lift on the way to other errands./ It is easier for me to take my child by car when I am on the way to other errands
• I have / my child has no desire to do so
• My parents do not allow me to do so.^a^

^a^only in the adolescents’ questionnaire

For 298 out of 364 children, information regarding all barriers as well as sociodemographic variables were available. The multivariate analyses were carried out on this complete subsample. The adolescent subsamples comprised 527 (out of 706, parent-reported information on barriers) and 570 (self-reported information on barriers) participants. Compared to participants without missing information, the excluded individuals had a significantly higher number of perceived barriers (b = 1.01, *p* = 0.02) in the children's subsample and in the parent-reported adolescent subsample (b = 1.71, p < 0.01). The number of self-reported perceived barriers was lower among the excluded participants in the adolescent subsample (b = 0.54, *p* = 0.02). Participants excluded from the children's subsample reported significantly less AST on the morning trip in the summer (Odds Ratio(OR) = 0.54, 95% confidence interval (CI) = 0.31–0.94, *p* = 0.025). Those excluded from the parent-report adolescent subsample reported longer distances between home and school ( b_100m_ = 8.57, *p* = 0.024). Participants excluded from the self-report adolescent subsample were younger than the included ones (b = -0.46 *p* = 0.032).

### Measures

SES was calculated as the Winkler Index, a composite score considering parental education, parental occupations, and equivalized household net equivalent income. Scores on the Winkler Index range from 3 to 21 [27]. As the share of participants with a low SES (index score 3–8.7) was very small, low and middle SES (index score 8.8–16.9) were combined into a single “low to middle” SES group.

The distance between home and school was calculated as the network distance from the home to school address using the Dijkstra algorithm [28]. Since our analyses showed a negative correlation between distance and AST only for pupils not living in the immediate sourroundings of their school (see results) we categorized distances into short distances (< 200 m for children, < 600 m for adolescents) and longer distances.

Information about means of transport (car, public transportation, walking, and/or cycling/skateboard/scooter), travel duration (minutes on each means of transport), and perceived barriers to AST were assessed in a questionnaire completed by the parents and, beginning at age 9.5, additionally by the children and adolescents themselves. Answers regarding the means of transport for participants below age 13.5 were primarily parent reports and only complemented with self-reports when parent reports were missing. For adolescents older than 13.5 years of age, self-reports were preferred because greater independence was assumed (Additional Fig. 1). The means of transport was reported separately for the way to school and the way from school back home, as well as for winter and summertime. A list of questions regarding the means of transport from the questionnaire for children and adolescents aged 9.5 and above is provided in Table 1. The questions in the parents’ questionnaire were structured in the same way.

**Fig. 1:**
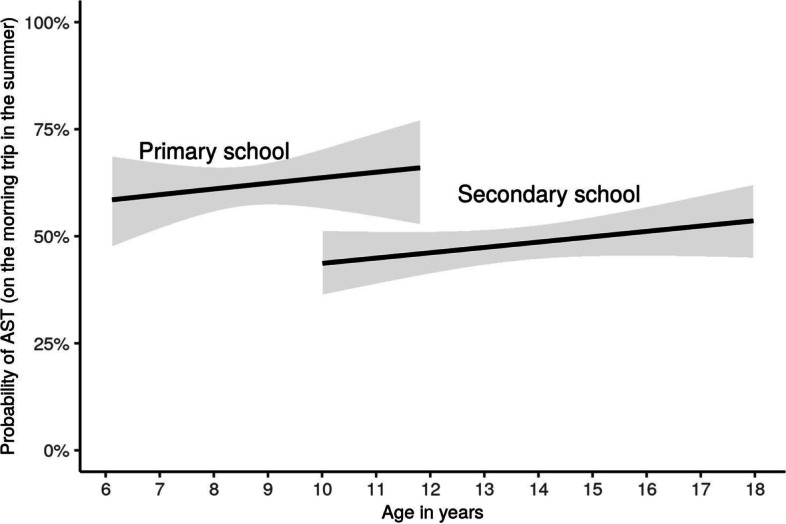
Associations between active school transport, age, and type of school: In general, a higher proportion of primary school children engage in active school transport. For both primary and secondary school children, the proportion increases with increasing age

Adolescents were additionally asked in a single-choice question to state which of the four given options they would prefer for their school transportation if they had free choice.

The primary means of transport was categorized as (1) cycling or (2) walking for children and adolescents who used cycling or walking as their only means of transport or who used a combination that included at least 15 min of cycling or walking. The third category public transport was assigned when participants took the bus, tramway, or train; the fourth category was being taken by car. Cycling and walking were considered active school transport (AST), whereas using public transport or being taken by car were considered motorized school transportation.

The perceived barriers were also assessed through questionnaires using the following phrasing:

“It is difficult for me / for my child to walk or to cycle to school independently (without the company of an adult) because…”. The participants could rate each potential barrier on a four-level Likert scale with the following options: “I disagree”, “I mildly disagree”, “I mildly agree”, and “I fully agree”. The first two answer options were aggregated into the category “non-significant barrier” and the last two answer options into the category “perceived as a potent barrier".

A list of questions and answers regarding the perceived barriers can be found in Table 2.

The phrasing of the questions is based on a Canadian research paper by Wilson et al. [29].

Furthermore, children and parents were asked to state whether or not bad weather conditions hinder them/their children from walking/cycling to school on a scale from 0–100 (with 0 = not at all and 100 = a lot). Ratings > 50 were interpreted as considering weather as a significant barrier to AST. For parents stating that they have no reservations about letting their children cycle or walk to school without an adult’s company, the perceived barriers were set to “no barrier”. The barrier “bullying” was removed from the analysis because of the low number of positive answers (n < 10). The number of perceived effective barriers was also considered as a covariate.

### Statistical analysis

All analyses were performed using the software R [30]. The prevalence of AST and the association between AST and SES were assessed separately for the morning and afternoon trip in summer and winter, respectively. The remaining analyses considered AST in the morning during summer as the primary outcome unless otherwise stated. Its relationship to sociodemographic variables was assessed using logistic regression models. Associations between AST and perceived barriers were estimated using multivariate logistic regression, stratified by the two age groups: children (≤ 10) and adolescents (> 10 years). Variable selection was based on the results of the univariate models (all hypothesized correlates: sociodemographic variables, perceived barriers, number of perceived barriers, interaction between distance and distance group).

In order to identify the final set of explanatory variables, all variables with a significant result in the univariate analyses were included in the multivariate modelling process. In a second step, following the statistical principle of parsimony, we used a forward and backward deletion algorithm based on the Akaike criterion and, thereby, removed stepwise all non-significant variables. Hence, the final models do not comprise all variables which were significant in the univariate analysis. The reported ORs are not adjusted except in the final models or when otherwise stated. We used Poisson regression models to compare the number of barriers perceived by parents of children, parents of adolescents, and adolescents themselves. The significance level was set to α = 0.05.

## Results

### Descriptive

On average, children lived 1930 m (SD: 2688) away from school and adolescents 3468 m (SD: 3210). The mean duration of the school trip was 13.28 min (SD: 8.84) for children and 19.50 min (SD: 13.30) for adolescents. AST was significantly more common in summer (morning: 53.64%, afternoon: 54.58%) than in winter (morning: 43.18%, afternoon: 44.49%, *p* < 0.01). Unless otherwise stated, the following results refer to the morning trip in the summer.

Sixty-three percent of the children (*n* = 54) and 57% of the adolescents (*n* = 403) stated that they would choose AST if they had free choice. In comparison, the number of children (59.34%) and adolescents (50.70%) who actually reported AST was lower. This difference was significant in the adolescent subgroup (*p* < 0.01), but not in the children subgroup (*p* = 0.576).

### Age, gender and SES

The share taking AST decreased with age (OR = 0.94 per year, CI = 0.9–0.99 *p* = 0.025). After controlling for school type, age exhibited a (non-significant) positive correlation with AST (Fig. 1).

Children in secondary school had significantly lower odds of AST than children in primary school (OR = 0.57, CI = 0.45–0.74, *p* < 0.01). When controlling for the distance between home and school, the effect of school type was inversed (OR = 1.41, CI = 1–1.99, *p* = 0.048), i.e., when considering the same distance, a secondary school child is more likely than a primary school child to use an active means of transport.

Walking was more frequently reported than cycling. However, the share of cyclers increased with age while the share of walkers decreased (Table 3).

**Table 3:** Distribution of means of transportation on the way to school in summer by age (values are % and (n))

		**By age**					
		**Children**			**Adolescents**		
**Means of transportation**	**total**	**6–8**	**8–10**	**10–12**	**12–14**	**14–16**	**16–18**
Bike	25.51% (273)	15.65% (41)	24.66% (55)	26.79% (60)	28.66% (47)	30.15% (41)	47.54% (29)
Car	16.17% (173)	25.95% (68)	28.25% (63)	8.48% (19)	6.10% (10)	5.88% (8)	8.20% (5)
Public transport	30.19% (323)	12.98% (34)	15.70% (35)	42.41% (95)	46.34% (76)	46.32% (63)	32.79% (20)
Walk	28.13% (301)	45.42% (119)	31.39% (70)	22.32% (50)	18.90% (31)	17.65% (24)	11.48% (7)

The prevalence of AST did not significantly differ by gender (children: OR_girls_ = 1.5, CI = 0.95–2.25, *p* = 0.075, adolescents: OR_girls_ = 1.01, CI = 0.75–1.37, *p* = 0.924).

**Fig. 2:**
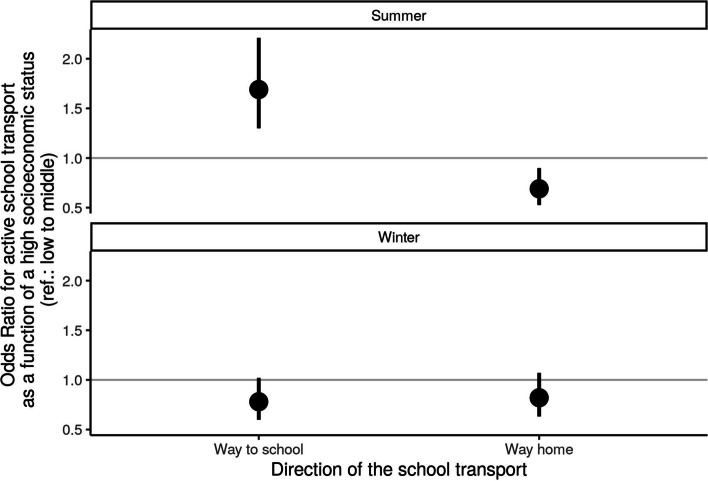
The correlation between socioeconomic status and active school transport differs by season and direction of travel: For both children and adolescents, the odds of AST decreased significantly as the distance between home and school increased (children: OR_100m_ = 0.90, CI = 0.88-0.93, *p* < 0.01; adolescents: OR_100m_ = 0.95, CI = 0.94-0.96, *p* < 0.01, Fig. 3)

Children and adolescents with a high SES had significantly higher odds of AST compared to those with a middle to low SES (OR = 1.69, CI 1.3–2.21, *p* < 0.01, Fig. 2). This effect persisted after controlling for distance. Interestingly, the association between AST on the way home (summer) with high SES was inverse OR = 0.69, CI = 0.53–0.9, *p* < 0.01). For the winter season, there was no significant correlation between SES and AST.

**Fig. 3:**
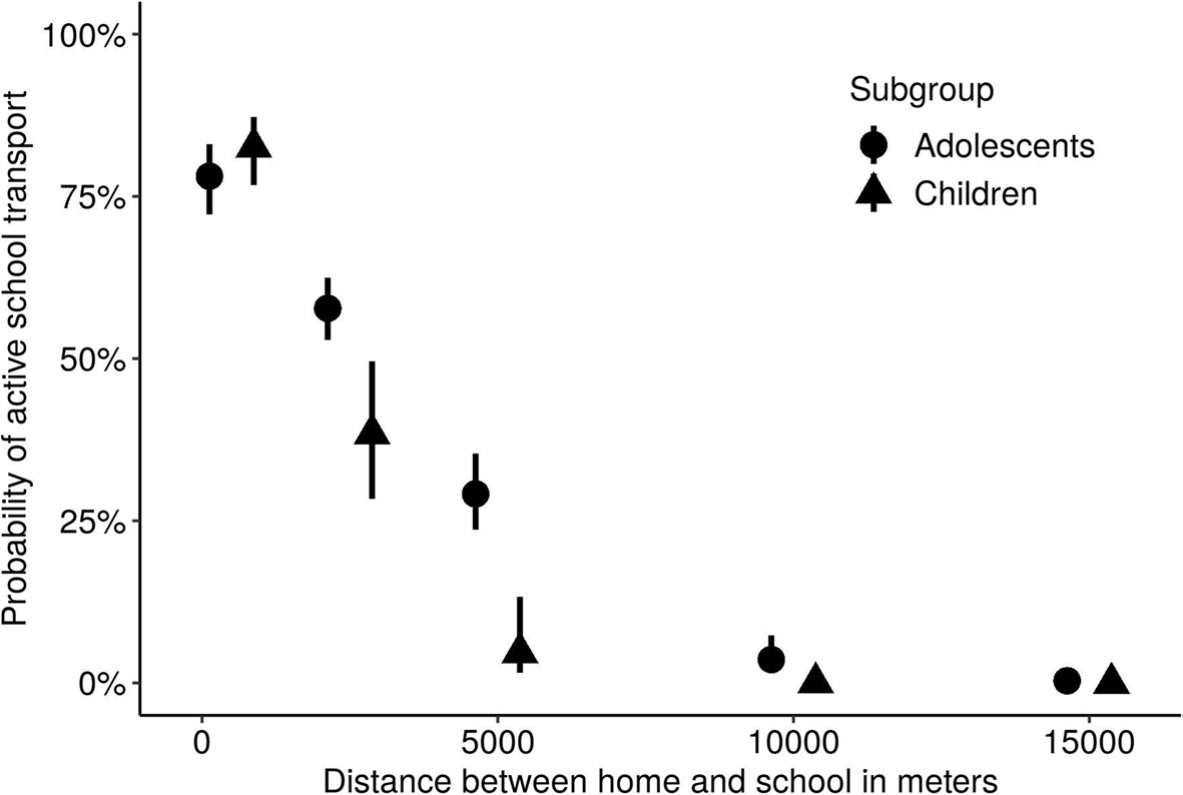
Association between active school transport and distance for children and adolescents: In both groups, the probability of active school transport decreased as the distance between home and school grows

For example, the estimated probability for AST was 85.97% for children living 250 m, 68.83% for children living 1500 m and 38.43% for children living 2500 m away from school. Likewise, for adolescents living 650 m away from school, the probability for AST was 76.86%, whilst it was 45.81% for adolescents living 3500 m away from school and 16.68% for adolescents living 6500 m away from school. For adolescents living close to their schools, there was a positive correlation between distance and AST (OR = 1.37, CI = 1.03–1.88, *p* = 0.025, *n* = 66). No significant relationship between distance and AST could be observed for children living close to their school.

### Perceived barriers

The mean number of perceived barriers was 3.26 (SD: 2.83) for the parents of children, 2.49 (SD: 2.86) for the parents of adolescents, and 1.55 (SD: 2.43) for adolescents themselves. The parents of adolescents perceived significantly fewer barriers than the parents of children (*p* < 0.01) and significantly more barriers than the adolescents themselves (*p* < 0.01).

The number of perceived barriers reported by parents and by adolescents showed significant inverse correlations with AST in the univariate models. In the univariate models for children ≤ 10 years, most barriers exhibited a negative correlation to AST. Only the way is too boring, too exhausting, and having no desire to walk/cycle were not associated with the outcome (Table 4).

**Table 4:** Models for children (≤ 10 years) analyzing correlations between active school transport and sociodemographic variables, distance between home and school, and perceived barriers (based on parental answers). OR = exp(ß)

Independent variable	OR univariate (95% CI)	*P*-value univariate	OR final (95% CI)	*P*-value final
**Socioeconomic status**				
Age	0.91 (0.73–1.12)	0.349		
Gender female (ref.: male)	1.47 (0.95–2.25)	0.075		
High socioeconomic status (ref.: low to middle)	1.55 (0.99–2.42)	0.051		
**Distance to school**				
Distance between home and school (in 100 m) for very short distances	0.91 (0.26–3.23)	0.88	1.02 (0.25–4.15)	0.972
Distance between home and school (in 100 m) for longer distances	0.90 (0.88–0.93)	< 0.01	0.90 (0.87–0.93)	< 0.01
**Perceived barriers**				
Number of perceived barriers	0.67 (0.60–0.74)	< 0.01		
**Physical environment**				
Too far	0.06 (0.03–0.12)	< 0.01		
Takes too much time	0.08 (0.04–0.14)	< 0.01		
Too hilly/too exhausting	0.00 (0.00-∞)	0.983		
No sidewalks or bike paths	0.37 (0.21–0.66)	< 0.01	2.53 (0.82–7.75)	0.098
Route too boring	0.34 (0.03–3.96)	0.379		
Insufficient street lighting	0.32 (0.16–0.63)	< 0.01		
**Safety**				
Route too dangerous	0.29 (0.18–0.45)	< 0.01	4.12 (1.33–12.80)	0.012
Crime	0.41 (0.21–0.78)	< 0.01		
Too much traffic	0.20 (0.12–0.32)	< 0.01	0.33 (0.11–1.00)	0.046
**Social**				
No other children	0.12 (0.07–0.20)	< 0.01	0.37 (0.14–1.00)	0.045
**Individual/Family preferences**				
Not enough confidence	0.38 (0.22–0.67)	< 0.01		
Too heavy load to carry	0.22 (0.14–0.36)	< 0.01	0.42 (0.18–0.94)	0.031
An adult gives a lift	0.09 (0.05–0.16)	< 0.01	0.09 (0.04–0.19)	< 0.01
No desire	0.95 (0.40–2.25)	0.913		

The answering options the path is too dangerous (positive correlation), too much traffic, no other children, too heavy load to carry*,* an adult giving a lift, and longer distance (only for children not living in the immediate surroundings of their school) also showed significant effects in the multivariate analyses (Table 4).

In the adolescent sample, the route being too exhausting or too boring were the only barriers not showing any significantly negative correlation with AST in the univariate analyses using the parental answers. The multivariate analysis using the parental answers showed significantly negative correlations between AST and the following perceived barriers: too heavy load to carry, number of perceived barriers, and longer distance (only for adolescents not living in the immediate surroundings of their school). Examining the adolescents’ answers, crime was the only barrier that did not show a significantly negative association with AST in the univariate models (Table 5). In the multivariate model, we found negative associations with it takes too much time, too much traffic*,* too heavy load to carry*,* bad weather, and distance (for adolescents not living in the immediate surroundings of their school). In contrast, for adolescents living in the immediate surroundings of their school, distance showed a significant positive correlation with AST (Table 5). Insufficient street lighting also revealed a significantly positive correlation, likely indicating generally insufficient infrastructure, including a lack of public transportation (Table 5).

**Table 5:** Models for adolescents (> 10 years) analyzing correlations between active school transport and sociodemographic variables, distance between home and school, and perceived barriers (separate models based on parental or adolescents' answers). OR = exp(ß)

	**Adolescents' answers**				**Parental answers**			
**Independent variable**	**OR** **univariate** **(95% CI)**	***P*** **-value univariate**	**OR** **final** **(95% CI)**	***P*** **-value** **final**	**OR univariat** **(95% CI)**	***P*** **-value univariate**	**OR** **final** **(95% CI)**	***P*** **-value final**
**Socioeconomic status**								
Age	0.98 (0.92–1.05)	0.6			0.98 (0.92–1.05)	0.6		
Gender female (ref.: male)	1.01 (0.75–1.37)	0.924			1.01 (0.75–1.37)	0.924		
High socioeconomic status (ref.: low to middle)	1.70 (1.21–2.38)	< 0.01	2.17 (1.34–3.49)	< 0.01	1.70 (1.21–2.38)	< 0.01	1.93 (1.07–3.48)	0.025
**Transport modes to school**								
Preferred means of transport active (ref.: passive)	10.10 (7.01–14.40)	< 0.01						
**Distance to school**								
Distance between home and school (in 100 m) for very short distances	1.37 (1.03–1.80)	0.025	1.34 (1.01–1.79)	0.040	1.37 (1.03–1.80)	0.025	1.37 (0.98–1.94)	0.064
Distance between home and school (in 100 m) for longer distances	0.95 (0.94–0.96)	< 0.01	0.96 (0.94–0.97)	< 0.01	0.95 (0.94–0.96)	< 0.01	0.95 (0.93–0.96)	< 0.01
**Perceived barriers**								
Number of perceived barriers	0.60 (0.53–0.66)	< 0.01			0.65 (0.59–0.72)	< 0.01	0.82 (0.70–0.96)	0.013
**Physical environment**								
Too far	0.05 (0.03–0.11)	< 0.01			0.06 (0.02–0.16)	< 0.01		
Takes too much time	0.08 (0.04–0.15)	< 0.01	0.22 (0.08–0.58)	< 0.01	0.06 (0.02–0.14)	< 0.01		
Too hilly/too exhausting	0.29 (0.14–0.60)	< 0.01			0.00 (0.00-∞)	0.979		
No sidewalks or bike paths	0.25 (0.13–0.48)	< 0.01	2.93 (0.85–10.01)	0.081	0.11 (0.05–0.26)	< 0.01		
Route too boring	0.43 (0.24–0.78)	< 0.01			0.20 (0.02–1.77)	0.139		
Insufficient streetlighting	0.27 (0.13–0.54)	< 0.01	4.37 (1.30–14.60)	0.015	0.19 (0.08–0.44)	< 0.01	6.33 (1.02–39.20)	0.043
**Safety**								
Route too dangerous	0.12 (0.05–0.30)	< 0.01	0.33 (0.10–1.12)	0.070	0.14 (0.07–0.28)	< 0.01		
Crime	0.57 (0.29–1.11)	0.092			0.16 (0.05–0.49)	< 0.01	0.22 (0.03–1.60)	0.126
Too much traffic	0.19 (0.11–0.31)	< 0.01	0.34 (0.15–0.74)	< 0.01	0.15 (0.08–0.27)	< 0.01		
**Social**								
No other children	0.17 (0.09–0.33)	< 0.01			0.12 (0.06–0.24)	< 0.01		
**Individual/Family preferences**								
Not enough confidence	0.13 (0.44–0.46)	< 0.01			0.28 (0.10–0.79)	0.014	6.05 (0.94–39.20)	0.054
Too heavy load to carry	0.19 (0.12–0.29)	< 0.01	0.43 (0.22–0.84)	0.012	0.10 (0.05–0.20)	< 0.01	0.13 (0.03–0.48)	< 0.01
An adult gives a lift	0.31 (0.17–0.58)	< 0.01			0.12 (0.05–0.27)	< 0.01		
No desire	0.26 (0.17–0.41)	< 0.01			0.10 (0.03–0.35)	< 0.01		
Parents do not permit it	0.12 (0.06–0.26)	< 0.01						
Bad weather conditions	0.39 (0.28–0.53)	< 0.01	0.48 (0.31–0.75)	< 0.01	0.14 (0.07–0.32)	< 0.01		

## Discussion

This study analyzed the prevalence of AST among children and adolescents and barriers perceived by parents and adolescents in a city of about 600 000 inhabitants in Germany. The prevalence of AST in our study sample (53.6%) ranges below the percentages published for Germany as a whole: 78.3% in a nationwide cohort of children and adolescents [14] and 64.8% in a sample of primary school children in southwestern Germany [31]. Another study found a prevalence of 44% (73% when also considering combined transport modes) among eight-year-old children in Leipzig [32]. This corresponds to the lower odds for active school transport in eastern Germany described by Reimers et al. [14]. Like a previous study from Norway, we found a lower prevalence of AST in winter [33], which can be attributed to more unfavorable weather conditions. However, comparisons between these studies are limited due to different cut-off points or definitions of AST.

Our data shows that more children and adolescents would engage in AST if they could choose than the number of children and adolescents who actually reported AST. This underlines the necessity of studying what external reasons deter children from AST. In our sample, the correlation between AST and age was negative but became positive when controlling for school type. The highest proportion was found at age 10, which is consistent with results of a Canadian study by Pabayo et al. [34]. We could show that the negative correlation with secondary school (vs. primary school) inverses when controlling for distance. This suggests that there is a higher potential for AST among secondary school pupils that remains unused due to the longer typical distance to secondary schools. The increasing cycling in older age groups is in line with previous findings from Germany [35] and may reflect the higher complexity and risk of cycling. Furthermore, bicycles are more appropriate for the longer distances to secondary schools. Whilst higher rates of AST among boys than girls have been described for Australia and the United Kingdom [21, 36], our results are consistent with the findings of a nationwide German study [14] and of a Turkish study [37] which found no meaningful gender difference in the prevalence. The literature is inconsistent regarding the relationship between SES and AST. For example, a German study reported a positive correlation between high SES and AST [31], whereas a study from the United Kingdom came to the opposite conclusion [38]. In turn, another study from the United Kingdom found a negative correlation between AST and living in a deprived area [39]. In our study, higher SES was associated with more AST in the morning trip and less AST in the afternoon (both in summer). A possible explanation for this might be conditions that favor AST, such as a walkable neighbourhood or the availability of a bicycle, in families with high SES. We hypothesize that parents with high SES are more likely to drive their children to fee-based afternoon activities like music lessons or sports clubs. Our analysis confirmed the importance of the distance between home and school, which has been found consistently in the literature [21, 40–43]*.* Previous studies concluded that school placement policies should aim to locate schools centrally in neighbourhoods to reduce the distance students need to travel [44, 45].

Regarding perceived barriers, significant associations were found in the domains of physical environment, safety, social and individual/family preferences. Taking too much time was a significant barrier for adolescents, but not their parents. Conversely, in a Canadian study, this was a barrier only perceived by parents [29]. Insufficient illumination showed a positive correlation with AST in both models for adolescents, likely indicating generally insufficient infrastructure like a lack of public transportation.

Counterintuitively, the route being too dangerous exhibited a positive correlation with AST in the model for children. However, again, this association might be attributable to worse infrastructure or a higher awareness of traffic-related dangers among parents of children who use AST out of necessity than among parents whose children use passive means of transport. Too much traffic exhibited a significant negative correlation in the self-reported model for adolescents and in the model for children, but not in the parent-reported model for adolescents. It can be assumed that the parents of adolescents are less familiar with the exact route and its characteristics due to adolescents’ higher degree of independence. Conversely, the number of barriers perceived by adolescents’ parents was a significant predictor of AST, suggesting that the number may be more decisive than any specific barrier. Previous studies have shown that traffic-calming measures can increase use of active transport to school and elsewhere [46, 47]. Parents of younger children perceived no other children walking or cycling as a significant barrier. Our findings thus confirm the results of a Canadian study, in which this aspect was described as significant from the point of view of parents, but not from the point of view of young people themselves [29]. This barrier might be addressed by implementing “walking school buses” or “bicycle trains”. Children can join a school- or parent-organized group of children walking or cycling to school together with an accompanying adult [48]. As walking school buses and cycling trains may reduce traffic around school, they can also contribute to abating the barrier too much traffic described above. Too heavy load to carry was the only barrier significantly related to AST for children as well as adolescents (self- and parent report). Therefore, reducing the weight of school bags is (still) a critical issue for promoting AST. Corresponding measures could include expanding possibilities to store learning materials like textbooks at school or employing electronic media like online textbooks. *A*n adult giving a lift while taking care of other errands was the strongest perceived barrier to AST for younger children. Therefore, choices for or against AST are not independent between household members. Convincing parents themselves to use active transportation, if possible, can promote AST in their children. Higher neighbourhood walkability is a determinant of AST as well as active commuting in adults [49, 50]. Such variables with an effect on both children’s and adults’ active mobility add an indirect effect to the direct effect on AST: adults using active means of transport cannot take their child to school in their car.

### Strengths and weaknesses

The strengths of our study include the objective definition of AST, which takes into account combined transport modes. To our knowledge, this is the only study assessing perceived barriers to AST for schoolchildren across the whole age span from 1^st^ to 12^th^ grade in Germany [35]. The differentiated consideration of two age groups creates an opportunity to better adapt future interventions to the respective target group*.* For example, the barrier no other children to walk with seems to be more relevant for parents of young children than for parents of adolescents, indicating that walking school buses can be particularly helpful for primary school students.

A weakness is that we could not control for possible cluster effects on the neighbourhood, school or family level, as the sample sizes per cluster did not permit this (e.g., the most frequented school in our sample accounted for less than 3% of participants). There was also a discrepancy in the phrasing of questions: The questions regarding barriers referred to independent active travel, whereas the transport mode question focused on the active/passive transportation mode, ignoring the aspect of independence.

Since our data collection was based on electronic case report forms, we were methodologically unable to survey children younger than 10 years, an age when they were not yet confident reading and using a computer. We also assume lower independence at that age. Therefore, the statements of the parents seemed to be of primary importance. Finally, the generalizability of our results might be limited because of the underrepresentation of participants with a low SES and because majority of participating families lived in an urban environment.

## Conclusions

Significant associations were found between AST and factors from the domains of physical environment, safety, social and individual/family preferences. Some of them were consistently negatively related to AST (e.g., a heavy load to carry*)* across all survey groups, whereas other differed (e.g., the route being too boring was significantly negatively associated with AST in adolescents but showed no associations when asking parents). These findings can provide many starting points for future interventions, especially since most children and adolescents would opt for AST if given a choice.

## Supplementary Information

**Additional file 1: Additional table 1.** Summary of studies comparing barriers to active school transport perceived by children/adolescents and their parents (without claiming completeness). For a systematic review on the state of research until 2014, see Lu et al. 2014[1].

**Additional file 2: Additional table 2.** Socioeconomic status by age group (values are n and (%)).

**Additional file 3.**

## Data Availability

The datasets on which the present study is based are not publicly accessible, since the publication of data was not covered in study participants’ informed consent. Potentially sensitive information is collected in the context of the LIFE Child study; therefore, the data protection concept requires that all researchers interested in accessing the data sign a project agreement. Researchers interested in gaining access to the data collected in the LIFE Child study may contact the committee on data use and access (dm@life.uni-leipzig.de, Ms. Yvonne Dietz).

## Abbreviations

AST: Active school transport
CI: 95% Confidence interval
OR: Odds ratio
SES: Socioeconomic status
SD: Standard deviation
