# Supplementary material for: Active school transport in an urban environment:prevalence and perceived barriers
Source: BMC Public Health. 2023 Mar 23;23:557. doi: 10.1186/s12889-023-15464-7 (PMC10037850; doi:10.1186/s12889-023-15464-7)
Supplement: Supplementary file 1 — Additional file 1: Additional table 1. Summary of studies comparing barriers to active school transport perceived by children/adolescents and their parents (without claiming completeness). For a systematic review on the state of research until 2014, see Lu et al. 2014[1]. [file 12889_2023_15464_MOESM1_ESM.docx]

*Additional table 1:* *Summary of studies comparing barriers to active school transport perceived by children/adolescents and their parents (without claiming completeness). For a systematic review on the state of research until 2014, see Lu et al. 2014**[1].*

| Study | Place | Study population | Main findings |
| --- | --- | --- | --- |
| Schönbach et al. 2020 [2]. | Germany, urban or suburban areas | 12- to 15-year-olds, their parents and teachers | "A “bicycle and related equipment,” the “way to school,” and “personalfactors” were reported needs, perceived by students and teachers of both genders and by mothers.Girls reported the additional gender-specific need for “social behavior in road traffic,” mothers and female teachers reported “role of parents,” and female teachers reported a “sense of safety.”“  Comment: The study focused on cycling as a specific form of AST and had a particular emphasis on comparisons between genders. |
| Ozbil et al. 2021 [3] | Istanbul, Turkey | Adolescents (12-14y) and their parents | „Parental barriers transcribed from the open-ended responses are in agreement with those of the students. The primary perceived barrier to AST is distance and secondary to this are traffic safety and crime.“ |
| Chillón P et al. 2014 [4] | USA (18 schools across the country) | 4th and 5th grade students and their parents | „..the most relevant significant predictors of active school travel were parent’s perceived barriers, specifically child resistance and safety and weather as well as the school’s percentage of Hispanic students.“ |
| Ikeda et al. 2019 [5] | Auckland, New Zealand | 8 to 13 years olds and their parents | "Parental perceptions of convenience, traffic safety and social interactions as well as child perception of neighbourhood safety were mutually associated with children’s AST. |
| Aranda-Balboa et al. 2021 [6] | Four Spanish cities | Children and adolescents and their parents  (girls’ mean age: 13.04 ± 1.89 years old; boys’ mean age: 13.02 ± 1.90 years old) | "Both children and adolescents perceived higher physical and motivational barriers and social support barriers towards ACS than their parents" "Additionally, the parents perceived higher distance, traffic safety, convenience, built environment, crime-related safety and weather as barriers towards ACS, than their children" |
| Pfledderer et al. 2021 [7] | A metropolitan area of Utah, USA | Primary school childern and their parents | "All parent and child perceptions of barriers to AC to school had low agreement."  " [...]more parent perceptions correlate with rates of AC when compared to child perceptions." |
| Onywera et al. 2018 [8] | Urban, suburban and rural areas in Kenya, Mozambique and Nigeria | Youth aged 10 to 12y and their parents | “Convergent validity for challenges/barriers to active transportation to school ranged from fair (0.30 - The route does not have good lighting) to substantial (0.77 - My child has a disability)“ and varied between different study sites“ |
| Wilson et al. 2018 [9] | Southwestern  Ontario, Canada | Elementary school children and their parents | "[…] parents perceive more barriers to AST than children, although both parents and children perceptions have an impact on AST behaviour."  "[...]social barriers were only significant when perceived by parents."  "[…] a combination of per-  ceptions on environmental, safety, social, and preference  barriers influence children AST." |

1. Lu W, McKyer ELJ, Lee C, Goodson P, Ory MG, Wang S. Perceived barriers to children’s active commuting to school: a systematic review of empirical, methodological and theoretical evidence. Int J Behav Nutr Phys Act. 2014;11:140.

2. Schönbach DMI, Vondung C, Hidding LM, Altenburg TM, Chinapaw MJM, Demetriou Y. Gender Influence on Students, Parents, and Teachers’ Perceptions of What Children and Adolescents in Germany Need to Cycle to School: A Concept Mapping Study. International Journal of Environmental Research and Public Health. 2020;17:6872.

3. Ozbil A, Yesiltepe D, Argin G, Rybarczyk G. Children’s Active School Travel: Examining the Combined Perceived and Objective Built-Environment Factors from Space Syntax. Int J Environ Res Public Health. 2021;18:E286.

4. Chillón P, Hales D, Vaughn A, Gizlice Z, Ni A, Ward DS. A cross-sectional study of demographic, environmental and parental barriers to active school travel among children in the United States. Int J Behav Nutr Phys Act. 2014;11:61.

5. Ikeda E, Hinckson E, Witten K, Smith M. Assessment of direct and indirect associations between children active school travel and environmental, household and child factors using structural equation modelling. Int J Behav Nutr Phys Act. 2019;16:32.

6. Aranda-Balboa MJ, Chillón P, Saucedo-Araujo RG, Molina-García J, Huertas-Delgado FJ. Children and Parental Barriers to Active Commuting to School: A Comparison Study. Int J Environ Res Public Health. 2021;18:2504.

7. Pfledderer CD, Burns RD, Byun W, Carson RL, Welk GJ, Brusseau TA. Parent and Child Perceptions of Barriers to Active School Commuting. J Sch Health. 2021;91:1014–23.

8. Onywera VO, Larouche R, Oyeyemi AL, Prista A, Akinroye KK, Heyker S, et al. Development and convergent validity of new self-administered questionnaires of active transportation in three African countries: Kenya, Mozambique and Nigeria. BMC Public Health. 2018;18:1018.

9. Wilson K, Clark AF, Gilliland JA. Understanding Child and Parent Perceptions of Barriers Influencing Children’s Active School Travel. BMC Public Health. 2018;18.
