# Supplementary material for: Active school transport in an urban environment:prevalence and perceived barriers
Source: BMC Public Health. 2023 Mar 23;23:557. doi: 10.1186/s12889-023-15464-7 (PMC10037850; doi:10.1186/s12889-023-15464-7)
Supplement: Supplementary file 2 — Additional file 2: Additional table 2. Socioeconomic status by age group (values are n and (%)). [file 12889_2023_15464_MOESM2_ESM.docx]

Additional *table 2*: Socioeconomic status by age group (values are n and (%)).

| Socioeconomic status | Children | Adolescents | Total |
| --- | --- | --- | --- |
| High | 150 (41.21%) | 214 (30.31%) | 364 (34.02%) |
| Middle | 187 (51.37%) | 431 (61.05%) | 618 (57.76%) |
| Low | 5 (1.37%) | 19 (2.69%) | 24 (2.24%) |
| Not available | 22 (6.04%) | 42 (5.95%) | 64 (5.98%) |
