# Supplementary figures and images for: Active school transport in an urban environment:prevalence and perceived barriers
Source: BMC Public Health. 2023 Mar 23;23:557. doi: 10.1186/s12889-023-15464-7 (PMC10037850; doi:10.1186/s12889-023-15464-7)

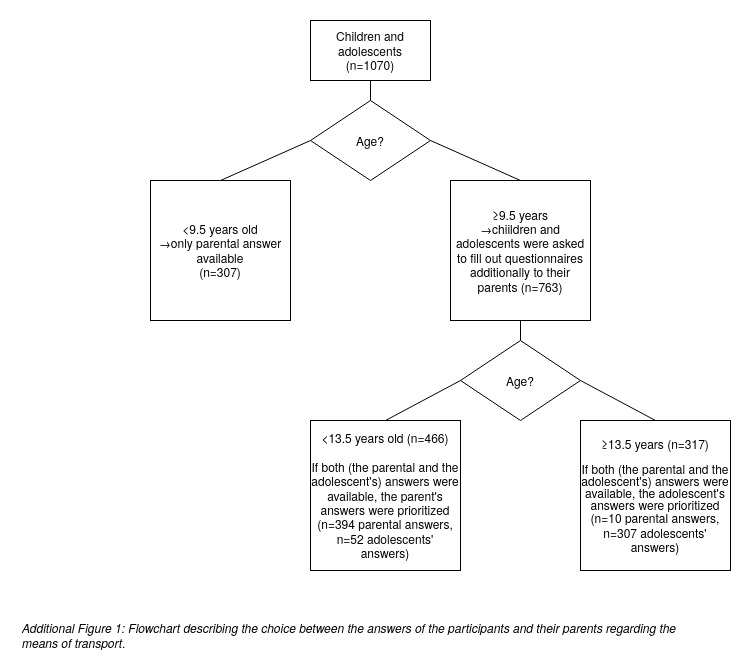

Supplement: Supplementary file 3 — Additional file 3. [file 12889_2023_15464_MOESM3_ESM.jpg]
